# Advantageous Inequity Aversion Does Not Always Exist: The Role of Determining Allocations Modulates Preferences for Advantageous Inequity

**DOI:** 10.3389/fpsyg.2018.00749

**Published:** 2018-05-23

**Authors:** Ou Li, Fuming Xu, Lei Wang

**Affiliations:** ^1^School of Management, Zhejiang University, Hangzhou, China; ^2^Neuromanagement Lab, Zhejiang University, Hangzhou, China; ^3^School of Psychology, Central China Normal University, Wuhan, China; ^4^School of Psychology, Jiangxi Normal University, Nanchang, China

**Keywords:** inequity aversion, fairness decision-making, advantageous inequity, SCRs, sense of agency, responsibility

## Abstract

Previous studies have shown that people would like to sacrifice benefits to themselves in order to avoid inequitable outcomes, not only when they receive less than others (disadvantageous inequity aversion) but also when they receive more (advantageous inequity aversion). This feature is captured by the theory of inequity aversion. The present study was inspired by what appears to be asymmetry in the research paradigm toward advantageous inequity aversion. Specifically, studies that supported the existence of advantageous inequity aversion always relied on the paradigm in which participants can *determine* allocations. Thus, it is interesting to know what would occur if participants could not determine allocations or simply passed judgment on *predetermined* allocations. To address this, a behavioral experiment (*N* = 118) and a skin conductance response (SCR) experiment (*N* = 29) were adopted to compare participants' preferences for advantageous inequity directly when allocations were *determined* and when allocations were *predetermined* in an allocating task. In the *determined* condition, participants could divide by themselves a sum of money between themselves and a matched person, whereas in the *predetermined* condition, they could simply indicate their satisfaction with an equivalent program-generated allocation. It was found that, compared with those in the *determined* condition, participants in the *predetermined* condition behaved as if they liked the advantageous inequity and equity to the same degree (Experiment One) and that the SCRs elicited by advantageous inequity had no differences from those elicited by equity, suggesting that participants did not feel negatively toward advantageous inequity in this situation (Experiment Two). The present study provided mutual corroboration (behavioral and electrophysiological data) to document that advantageous inequity aversion may differ as a function of the individual's role in determining allocations, and it would disappear if individual cannot determine allocations.

## Introduction

Equity is a fundamental concern in people's interactions that influences many aspects of daily life, from how people share their resources with partners to how policymakers shape income distribution policy. A key component of equity is related to inequity aversion, which means that individuals resist inequitable outcomes; that is, they are willing to give up some material payoff to move in the direction of more equitable outcomes (Fehr and Schmidt, 1999; Bolton and Ockenfels, 2000), not only when they receive less than others (i.e., disadvantageous inequity aversion, DI) but also when they receive more (i.e., advantageous inequity aversion, AI). It is well-accepted that inequity aversion captures the critical feature of humans' fairness in decision-making (Fehr and Schmidt, 2006; Tricomi and Sullivan-Toole, 2015). Its empirical applicability has been confirmed not only by several subsequent experiments conducted by Ernst Fehr et al. (Falk et al., 2003; Knoch et al., 2006; Fehr et al., 2008) but also by other researchers from the fields of psychology (Blake and McAuliffe, 2011; Güroglu et al., 2011), economics (Eckel and Grossman, 2001; Fershtman et al., 2012), anthropology (Henrich et al., 2001), neuroscience (Sanfey et al., 2003; Tricomi et al., 2010; Tricomi and Sullivan-Toole, 2015), and other disciplines.

Given that inequity aversion is the main theory for understanding humans' fairness behaviors and can even be seen as the preferred approach to explore this issue (Xu et al., 2016), an in-depth analysis of inequity aversion seems essential. We believe that at least one deficiency has remained unsolved in the current research; that is, the research paradigm toward AI is asymmetric. Currently, studies on AI always have the participants themselves decide how to divide some resources between themselves and others and use the proportion that they share as the measure of their degree of AI (Tricomi and Sullivan-Toole, 2015; Xu et al., 2016)^1^. Studies using this paradigm have found that the majority of participants would offer 40–50% of the total sum to others (see a meta-analysis: Oosterbeek et al., 2004; or a review: Güth and Kocher, 2014). Therefore, they claimed that people have a strong preference for equity instead of for self-interest. However, when considering this paradigm, we can easily find an inherent feature that may weaken the reliability of such a conclusion; that is, all of the final offers in this paradigm are the results of the self-executed actions of participants. That is, participants can determine allocations on their own initiative. For example, a participant considers how to divide a sum of 10 RMB; he can keep all for himself (10, 0), divide the sum equitably (5, 5), or choose any amount *x* in the range of 10 (*x*, 10-*x*) (henceforth, the number on the left is given to the participant, while the number on the right is given to the other). For this, it is implied that current studies, most of which support the existence of AI, have relied solely on the paradigm in which participants can *determine* allocations while ignoring the paradigm in which they cannot.

Given this asymmetry, it is interesting to know what would occur if participants could not determine allocations or simply passed judgment on *predetermined* allocations. Indeed, to date, few studies have involved *predetermined* allocations or inactions. For example, in Albrecht et al.'s (2013) design, participants were required to indicate their satisfaction with a series of allocations that were assigned by experimenters [including an advantageous one, i.e., (20, 30)]. Furthermore, in Moser et al.'s (2014) and Lamichhane et al.'s (2014) ultimatum game task, participants were placed in the role of the responder instead of in the general role of proposer, such that they could merely say “yes” or “no” to an allocation (advantageous, equitable, or disadvantageous) imposed by the opponent but could not determine how to divide the offer. All of these studies found that people in such a situation appeared to prefer advantageous inequity, which conflicted with the theory of inequity aversion. Although these studies gave some insights on the open questions, the paradigm feature of *determined* or *predetermined* was not at their center^2^, and they also failed to manipulate it. Therefore, it is still unclear whether the individual's role in determining allocations could affect their AI degree. To address this, the present study may be the first to manipulate the paradigm feature of *determined*/*predetermined* and investigate its effect on AI.

In the area of decision-making, the individual's reaction to outcomes following their actual actions may be different from reactions to outcomes following inactions. For example, Kahneman and Tversky (1982) found that negative outcomes resulting from actions induced more regret than the same outcomes resulting from inactions. Such an effect can also be manifested by the omission bias, i.e., the tendency that people are more likely to judge harmful actions as worse or less moral than equally harmful inactions (Ritov and Baron, 1990). Subsequently, Choshen-Hillel and Yaniv (2011, 2012) extended this action effect to the area of prosocial preference by showing that the sense of agency, which was defined as “a person's degree or level of control over her or his outcomes and those of other parties” in their publication, can increase one's concern with another's welfare. Considering outcomes (11, 10) and (10, 11), in one of their experiments, 26.7% of the participants in the high-agency group chose the other-dominated outcome (10, 11), even at a financial cost to themselves. In contrast, only 6.5% of the participants in the low-agency group chose the same outcome. For a decision-maker, since an equitable allocation (vs. an advantageous allocation) is more in the interest of others, he may less frequently maintain equality when he cannot control outcomes than when he has the ability to do so. Choshen-Hillel and Yaniv (2011) considered that those who had a higher agency might view the others' outcomes as evidence of their own effectiveness and generosity and derive positive utilities from these outcomes. This idea is connected with the finding that the fact of having a choice itself can activate the subjective reward processing (for a review, see Leotti et al., 2010). For example, Leotti and Delgado (2011) found that merely anticipating an opportunity for choice could recruit the reward-related brain circuity, particularly the striatum. It is possible that the internal reward resulting from actual actions can partly offset the cost of giving to others, making individuals who have control more likely to be kind to others. From this review, it is suggested that the actual actions (i.e., *determining* an allocation on one's own initiative) could make people's focus change from self-interest to the other's welfare. Inaction (i.e., passively receiving a *predetermined* allocation), in contrast, would lead to the opposite effect. Taken together, the first hypothesis is that individuals' role in determining allocations can modulate their preference for advantageous inequity:

*Hypothesis 1:* Participants would show a strong tendency of AI in the *determined* condition, as previous studies claimed, whereas their tendency of AI would diminish or even disappear in the *predetermined* condition.

Previous studies have indicated the importance of negative emotions in inequity aversion (Xu et al., 2016). When participants received an inequitable allocation, their self-reported negative emotional responses, such as anger, spite, or sadness, increased (Pillutla and Murnighan, 1996; Bosman et al., 2001). These correlations were also manifested by neuroimaging studies. For example, Harlé et al. (2012) and Sanfey et al. (2003) found that the anterior insula, a brain region specifically involved in representing negative emotional states, played a critical role in processing inequitable outcomes. Its activation degree could also be used to predict the likelihood of someone's acceptance or rejection of inequitable allocations (Tricomi and Sullivan-Toole, 2015). Therefore, the arousal of negative emotions can be an indicator of the aversion toward advantageous inequity in the present study. The skin conductance response (SCR) is a measurement of the electrical conductance of the skin. It is related to physiological arousal elicited by the cognitive inhibition system (Fowles, 1980), which, in turn, is supposed to be the biological basis of negative emotions (Gray, 1994) and is commonly used as an electrophysiological indication to evaluate the feeling of inequity/unfairness (Tricomi and Sullivan-Toole, 2015). Indeed, there is growing evidence that an increased SCR is positively correlated with an increased degree of inequity that one is exposed to and an increased negative feeling that one is experiencing (van't Wout et al., 2006; Civai et al., 2010; Hewig et al., 2011; Dunn et al., 2012). Analyzing SCRs to advantageous inequity in the *determined/predetermined* conditions can provide more convincing evidence on the present issue. Based on the aforementioned reviews, we formed the second hypothesis that SCRs elicited by advantageous inequity can be modulated by the *determined/predetermined* feature during allocation:

*Hypothesis 2:* Advantageous inequity (vs. equity) might elicit a higher SCR in the *determined* condition, (i.e., a strong feeling of inequity), whereas the SCR elicited by the equivalent advantageous inequity may be the same as that elicited by equity.

To sum up, the main object of the present study was to investigate whether individual's preferences for advantageous inequity was affected by their role in determining allocations. More specifically, would they still resist advantageous inequity, or would they like it if this inequity did not result from their actions but was *predetermined*? To test this, we conducted a behavioral study (Experiment One) and a SCR study (Experiment Two) in the money distribution setting. In the *determined* condition, participants could decide by themselves how to divide a sum of money between themselves and a matched person, following the same procedure adopted by most studies (Fehr and Schmidt, 2006). In the *predetermined* condition, participants were asked to indicate whether an equivalent program-generated allocation between themselves and the match would satisfy them; in particular, they could not determine the allocation. Across the *determined*/*predetermined* conditions, the difference between participants' role in determining allocations was salient, while all other aspects between conditions were constant.

## Experiment one

### Materials and methods

#### Participants

In total, 141 college students, who were anonymous to each other, were recruited in this experiment. Seven participants were excluded because they did not believe that they had performed the task together with a real person simultaneously (they rated below 4 points on a 7-point Likert scale presented after the experiment; the remaining participants rated 5.856 ± 0.989 points on average). In addition, participants who majored in psychology or economics (seven and nine, respectively) were also excluded. With this inclusion criterion, 118 participants aged 18–23 years old (on average, 19.57 ± 0.70 years) were finally left (45 males, 73 females). This experiment was approved by the research ethics board of Central China Normal University. Informed consent was obtained from all participants before the experiment.

#### Study design

A 2 (Condition: *determined* vs. *predetermined*) × 5 [Allocation: (8, 2) vs. (7, 3) vs. (5, 5) vs. (3, 7) vs. (2, 8)] mixed design was employed, with the Condition referring to a between-subject factor and the Allocation referring to a within-subject factor. Thus, participants were randomly assigned to either the *determined* or the *predetermined* condition. Advantageous offers could be (8, 2) or (7, 3), the equitable offer was (5, 5), and disadvantageous offers could be (3, 7) or (2, 8). To make the distributions continuous, the present design also included the offers (6, 4) and (4, 6). In the past, a large body of studies have found that people tend to view an offer that is 10% over or under the median (i.e., 40–60%) as being reasonable (Camerer, 2003; Güth and Kocher, 2014). In other words, whether it is (6, 4) or (4, 6), people view it as a form of marginally equitable offers, although there is still some objective disparity. Because of this, (6, 4) and (4, 6) are not clear-cut: some decision-makers may see they as equitable, while others may disagree, producing mixed results overall. To clarify the difference between equity and inequity, studies of inequity aversion commonly exclude those confused offers from their design or analysis, as did Sanfey et al. (2003) and (van't Wout et al., 2006). The offers (6, 4) and (4, 6) therefore were included as filler tasks in the present study. Furthermore, (10, 0), (9, 1), (1, 9), and (0, 10) were excluded because these cases were too extreme to be chosen by real people and were unusual in daily life (Güth et al., 1982; Falk et al., 2003). The outcome factor was participants' preference for different Allocations, namely, the degree of inequity aversion.

#### Experimental procedure

The participants completed experimental tasks collectively in a standard laboratory. Since the capacity of the laboratory was up to 43, we had to conduct four experiments successively, three of which were held in January 2017 and one in May. The participants were randomly assigned to one of four sessions. Using the collective measure has two advantages: first, it can equalize the external situation (such as time, temperature, and brightness) imposed on each participant; second, this allowed us to easily manipulate participants' belief that “I am completing the task with someone else simultaneously.” We wanted to make participants believe that they played the task with a real person randomly selected from the same room at the same time because this could arouse their real motivation in decision-making. Actually, this was a deceptive operation, and the response of the alleged partners was set by experimenters (we debriefed participants at the close of the experiment about the true nature of the research).

**Figure 1:**
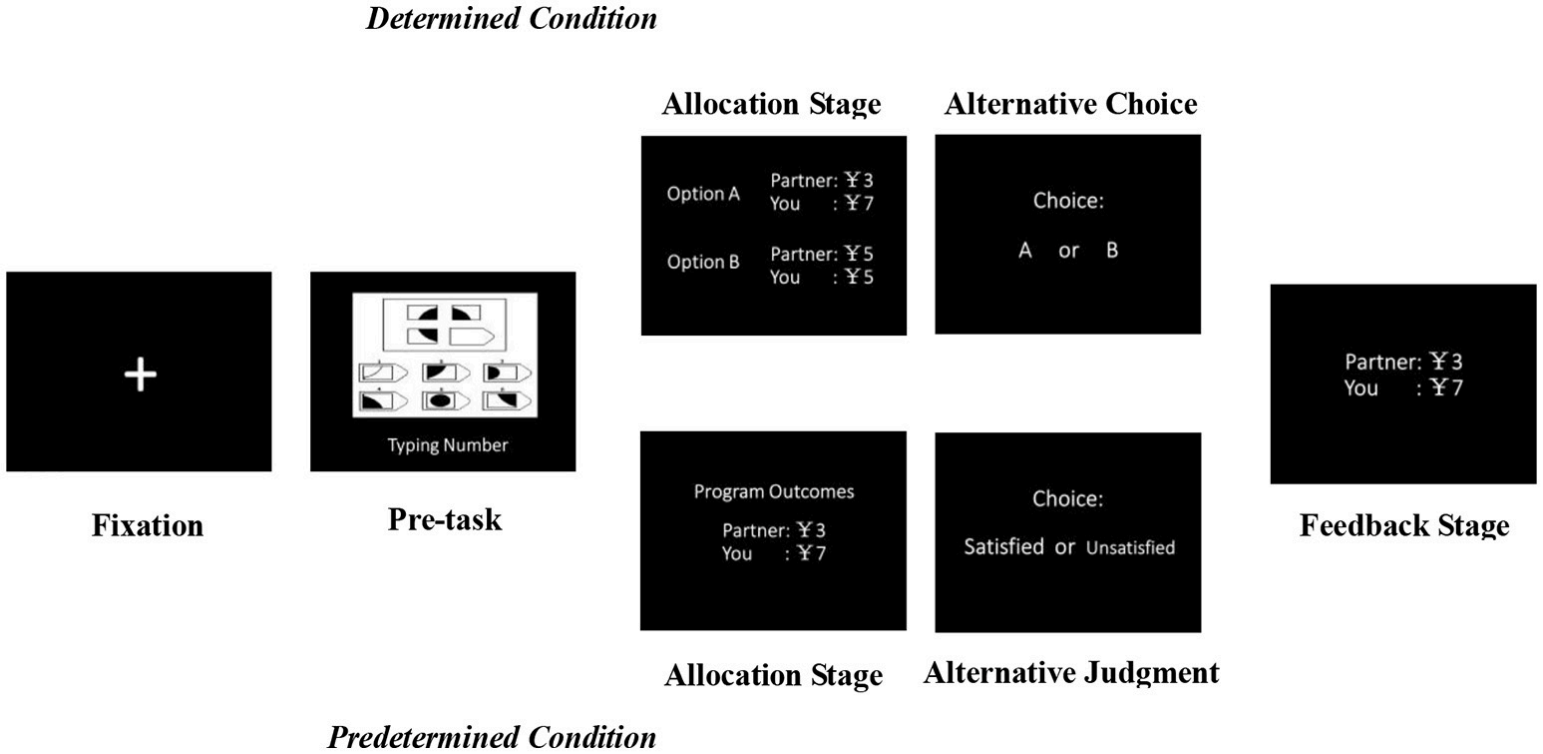
Experiment One procedure. Participants were randomly assigned to either the *determined* or the *predetermined* condition. One participant and a matched person attended the experiment, and all of them first completed a pre-task picked from Raven Matrices. Then, the participant was asked to consider the scheme on how to divide a reward of 10 RMB. In the *determined* condition, he had to make a two-alternative forced choice to divide the reward. In the *predetermined* condition, he had to make an alternative satisfaction judgment to a program-generated offer. Afterwards, payoffs for both the participant and the match in that round were presented at the Feedback Stage.

All tasks were conducted by computer. As illustrated in Figure 1, at the beginning of each trial, a fixation appeared as a cue for 3,000 ms on the black screen, which was followed by a pre-task. As the very definition of inequity is receiving uneven outcomes despite investing the same effort (Adams, 1965), we decided to use a pre-task so that the effort of both sides could be balanced out. Before the experiments, five psychology graduates were recruited to select pre-tasks from Raven Matrices, which ensured that the chosen pre-task was simple enough and could not impact the following tasks. According to the check beforehand, all pre-tasks (*n* = 14) were easy to solve (on average, 2.017 ± 0.913 points on a 7-point Likert scale for difficulty), and there was no significant difference between scores [*F*_(13, 117)_ = 1.004, *p* = 0.452]. A further test showed that the pre-tasks had no effect on the later responses [*F*_(5, 928)_ = 0.294, *p* = 0.917], and neither did the interaction effect of the pre-tasks and Condition [*F*_(2, 928)_ = 0.15, *p* = 0.861]. After the pre-task, the participant and the matched person could receive a reward of 10 RMB for their completion of the task, and then the participant was asked to consider the scheme on how to divide this reward between himself and the matched person.

In the *determined* condition, the participant had to make a two-alternative forced choice between an always equitable offer (5, 5) and an always inequitable offer, which was either advantageous or disadvantageous (Allocation Stage and Alternative Choice). Participants' preference was counted as their choice rate for each offer. Contrary to the *determined* condition, in the *predetermined* condition, a random program-generated offer from one of the five Allocations (Allocation Stage) was first presented, and then participants were asked to make a two-alternative forced judgement of whether they were satisfied with the offer (Alternative Judgment). Thus, participants' preference was counted as their satisfaction rate for each offer. Importantly, the revealed preference theory assumed that the preference of a decision-maker could be revealed by his actual decision-making, suggesting that one would choose the thing that satisfies him most (Samuelson, 1938). From this perspective, the choice that people makes is whatever they are satisfied with, thus establishing a connection between the alternative choice of the *determined* condition and the alternative judgment of the *predetermined* condition. Afterwards, the Feedback Stage lasted until participants pressed the Enter key at the end of each trial.

Participants' final income was related to the actual outcome of their decision-making in each trial, which was paid in the ratio of 12:1 (each participant gained 5.75 ± 0.73 RMB on average, added to a show-up fee 5 RMB). This allowed us to simulate the real-life situation in which individuals are remunerated for their work, providing the participant with a meaningful basis for comparing their own and the matched partner's incomes.

The design offer and the filler task offer were repeated twice, preceded by a practice session, and the presentation order of all trials was randomized by the program. Stimuli, recording triggers, and responses were presented adopting E-Prime 1.0 software package (Psychology Software Tools, Pittsburgh, PA, USA).

### Results

Statistical analysis was conducted in SPSS 23.0. The Greenhouse-Geisser correction for violation of the assumption of sphericity was applied when necessary. The Bonferroni correction was used for pairwise comparisons. We excluded (4, 6) and (6, 4) from the analysis because they were filler tasks. Nevertheless, we still take them into consideration in an additional test; for results see Appendix A.

Preferences for each Allocation across Conditions are presented in Table 1. Gender had no effect on the preference [*F*_(1, 114)_ = 0.092, *p* = 0.762], and neither did the interaction effect of gender and Condition [*F*_(1, 114)_ = 1.460, *p* = 0.229]. Similarly, there were no effects of the order of experiments [*F*_(3, 110)_ = 0.213, *p* = 0.887] or of the interaction between the order and Condition [*F*_(3, 110)_ = 0.200, *p* = 0.896].

**Table 1:** The descriptive data of the preference for each Allocation in the *determined* and *predetermined* conditions.

**Inequity types**	**Allocations**	**Preference in the *determined* condition (%) (*N* = 59)**	**Preference in the *predetermined* condition (%) (N = 59)**	**Facilitating Effects (%)**
Advantageous	(8,2)	13.56 ± 31.94	88.14 ± 31.26	72.6
	(7,3)	18.64 ± 38.17	88.98 ± 29.46	
Equitable	(5,5)	88.56 ± 19.32	94.07 ± 18.77	5.51
Disadvantageous	(3,7)	7.62 ± 20.94	25.42 ± 39.80	19.49
	(2,8)	5.93 ± 20.92	27.11 ± 39.74	

*Facilitating effects are the variations of preferences with the Condition changing from determined to predetermined, which was calculated as the positive change between conditions*.

The repeated measure ANOVA for preferences yielded main effects of both Condition [*F*_(1, 116)_ = 140.04, *p* < 0.001, η^2^ = 0.547] and Allocation [*F*_(2, 273)_ = 147.18, *p* < 0.001, η^2^ = 0.559], and further yielded an interaction effect of the two factors [*F*_(1, 116)_ = 39.22, *p* < 0.001, η^2^ = 0.253]. The results of the simple effect analysis for Condition showed that participants were more satisfied with all inequitable offers in the *predetermined* condition than in the *determined* condition [(8, 2): *p* < 0.001; (7, 3): *p* < 0.001; (3, 7): *p* < 0.01; (2, 8): *p* < 0.001, respectively]. However, preferences for the equitable offer (5, 5) were not significantly different (*p* = 0.119). More importantly, the results of the simple effect analysis for Allocation indicated that this factor had significant effects in both the *determined* [*F*_(4, 464)_ = 92.26, *p* < 0.01] and the *predetermined* [*F*_(4, 464)_ = 94.13, *p* < 0.001] conditions. Because of this, we would examine them respectively below.

**Figure 2:**
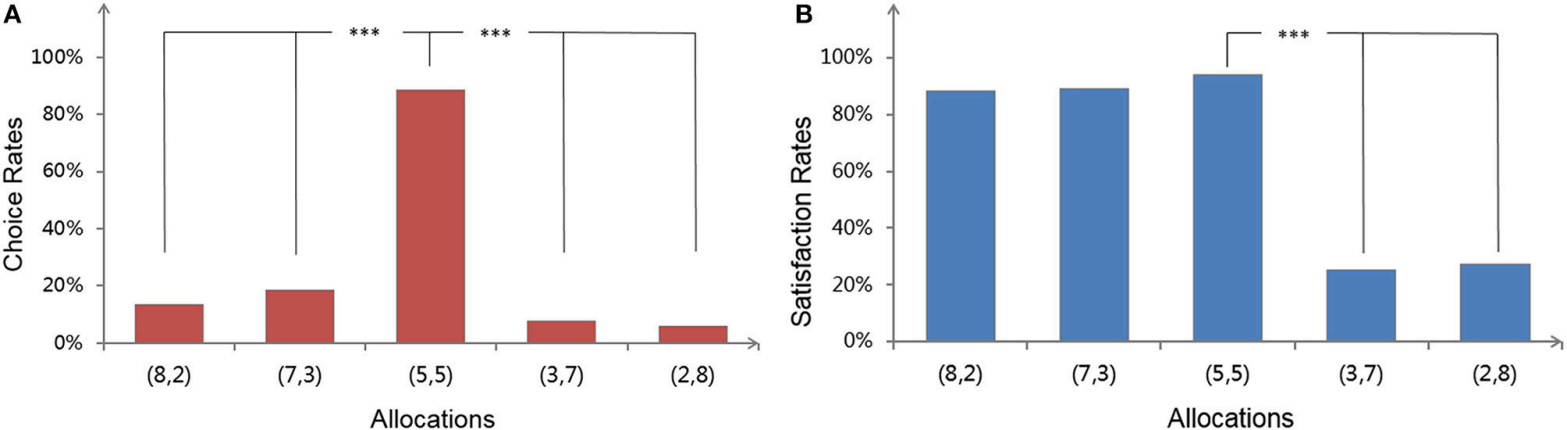
Effects of Allocation on preferences in the *determined* (left, **A**) and *predetermined* condition (right, **B**). Significant differences (*p* < 0.001) between Allocations are marked with ^***^.

In the *determined* condition, as shown in Figure 2A, pairwise comparisons showed that participants were more willing to choose (5, 5) compared to inequitable offers, regardless of whether the inequitable offers were advantageous or disadvantageous. For inequitable offers, only one pair reached statistical significance, with (7, 3) having a higher response than (2, 8) (*p* < 0.05). In addition, no other pairs had a significant difference. However, in the *predetermined* condition, the case was totally different. As illustrated in Figure 2B, pairwise comparisons showed that the satisfaction judgment rates for advantageous offers (8, 2) and (7, 3) were not different from the rates for the equitable offer (5, 5), while the rates for both were significantly higher than disadvantageous offers (3, 7) and (2, 8).

### Discussion

The results of Experiment One supported Hypothesis 1. More specifically, participants resisted receiving more than others only when they could determine allocations, which was consistent with previous studies (Fehr and Schmidt, 1999, 2006). However, once they simply passed judgment on *predetermined* allocations, they became satisfied to find that they had a higher payoff than others, which implied that their tendency of AI might disappear. It is noted that the overall preferences for inequitable offers were higher in the *predetermined* condition (vs. *determined* condition), which suggested a facilitating effect of making participants become more accepting of inequitable outcomes with the change of the task feature from *determined* to *predetermined* (see Table 1). By conducting a *t*-test for this between (dis)advantages, we found that its influence on advantageous inequity was nearly four times as great as that on disadvantageous inequity [72.46% vs. 19.49%, *t*_(234)_ = 8.953, *p* < 0.001, Cohen's *d* = 1.426], suggesting that participants would sharply turn from resisting advantages to seeking advantages as long as their control in the allocating was removed.

## Experiment two

### Materials and methods

#### Participants

In total, 31 healthy right-handed college students (10 males, 21 females), whose major was neither psychology nor economics, were recruited in this experiment; 27 of them had never joined a psychological experiment before. The ages of the participants ranged from 18 to 23 years (on average, 19.52 ± 0.65 years). All of the participants were included in behavior analysis, while three participants were excluded from SCRs analysis because they were outliers according to the boxplot. The final income a participant could earn equaled the sum of a show-up fee of 15 RMB and a performance-based fee, which was similar to that of Experiment One, although the paid ratio increased to 8:1. This experiment was approved by the research ethics board of Central China Normal University. Informed consent was obtained from all participants before the experiment.

#### Study design

Similar to Experiment One, a 2 (Condition: determined vs. predetermined) × 7 [Allocation: (5, 1) vs. (5, 2) vs. (5, 3) vs. (5, 5) vs. (5, 7) vs. (5, 8) vs. (5, 9)] mixed design was adopt, with the Condition referring to a between-subject factor and the Allocation referring to a within-subject factor. Thus, participants were randomly assigned to either the *determined* or the *predetermined* condition. Importantly, in a prior test, we found that SCRs were susceptible to the absolute payoff that one received in an allocation. Thus, the design was changed from amount-constant to payoff-constant, whereby participants' payoffs were kept constant at five units in each trial. Correspondingly, (5, 1), (5, 2), and (5, 3) were assigned to advantageous inequity, (5, 5) was designated to equity, and (5, 7), (5, 8), and (5, 9) were assigned to disadvantageous inequity. To control the confounding effect of the variation of amount, we did not present allocations in the form of showing payoffs for two players directly, such as “You: 7 RMB, Partner: 3 RMB.” Instead, we told participants the positive or negative difference between their payoff and their partner's payoff, such as “You get 5 RMB, Your partner gets 2 RMB less (more) than you.” The outcome factors were preferences and SCRs elicited by different Allocations.

#### Experimental procedure

Participants completed experimental tasks with a matched person in a quiet laboratory. In this experiment, we told the participants that our research focused on their numerical ability, preventing them from conjecturing our real purposes. Therefore, the pre-task in this experiment was replaced by a correlation judgment task, in which participants needed to evaluate the correlation coefficient from a scatter plot. Before the experiments, five psychology graduates were recruited to design the correlation judgement task, which ensured that the chosen pre-task was simple enough and could not impact the following tasks. According to the check beforehand, all pre-tasks (*n* = 16) were easy to solve (on average, 1.875 ± 0.815 points on a 7-point Likert scale of difficulty), and there was no significant difference between the scores [*F*_(15, 135)_ = 1.153, *p* = 0.316]. A further test showed that the pre-tasks had no effect on the following responses [behavior data: [*F*_(8, 435)_ = 0.305, *p* = 0.964], SCRs data: (*F*_(8, 405)_ = 0.374, *p* = 0.934)], and neither did the interaction effect of the pre-tasks and Condition [behavior data: [*F*_(8, 928)_ = 0.319, *p* = 0.959], SCRs data: (*F*_(8, 405)_ = 0.336, *p* = 0.952)]. Indeed, the matched person in this experiment was an experimental confederate, who was a female graduate student and a stranger to all of the participants. After the real participant came to the laboratory, followed by the confederate, he/she and the confederate were told that they would sit in front of two computers face to face and perform a task together through the computer network. The real participant was ostensibly selected by lot to the position required for the experiment. According to the survey after the experiment, no participants doubted this manipulation, and on average, they rated 8.656 ± 0.135 points on a 9-point Likert scale.

**Figure 3:**
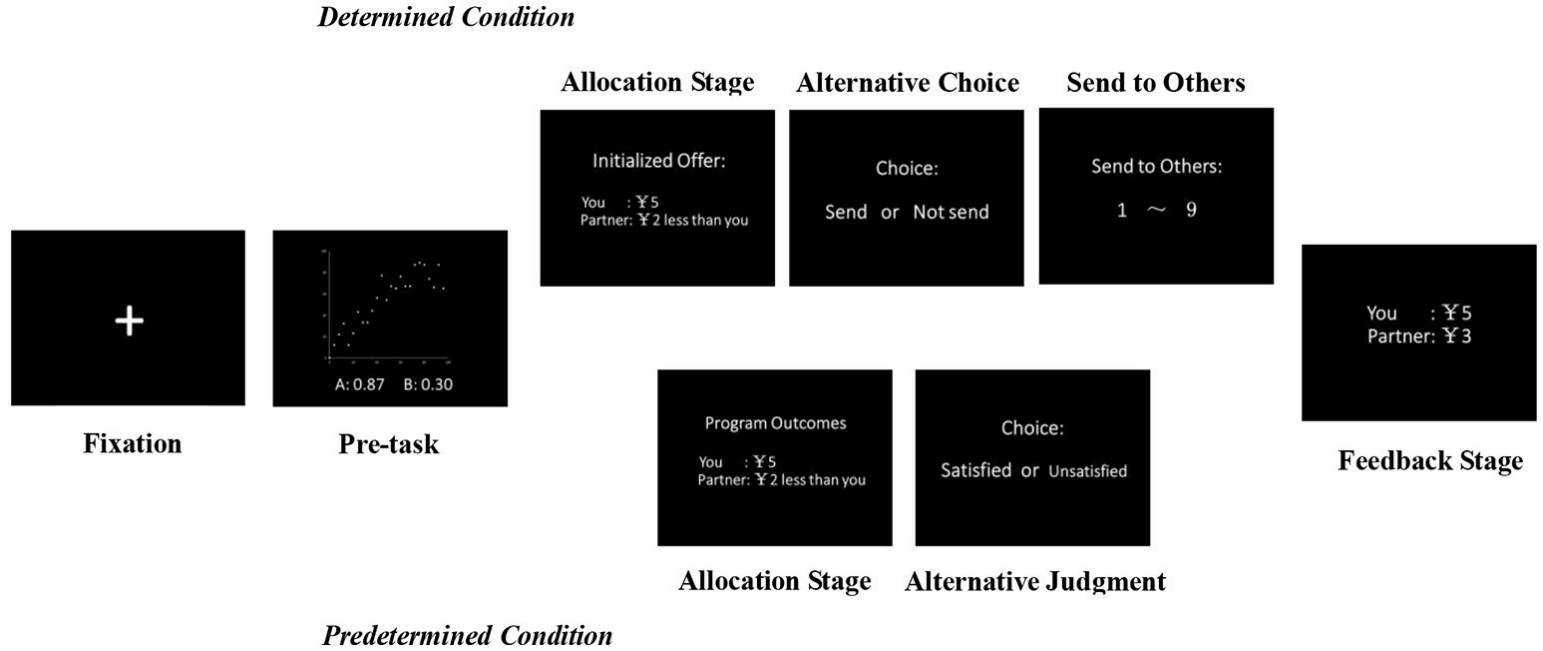
Experiment Two procedure. Participants were randomly assigned to either the *determined* or the *predetermined* condition. A participant and a matched person attended the experiment, and both of them first completed a pre-task (correction judgment task). Then, the participant was asked to consider the scheme on how to divide a reward. In the *determined* condition, he had to make a two-alternative forced choice of whether to send a presented offer to the match. If he approved, then the final distribution was assigned as the offer; otherwise, he could determine the unit he wanted to send to the match. In the *predetermined* condition, participants had to make an alternative satisfaction judgment to a program-generated offer. Afterwards, payoffs for both the participant and the match in that round were presented at the Feedback Stage. SCRs were recorded from the participant throughout the experiment.

The main procedure was similar to Experiment One. All tasks were conducted by computer. As illustrated in Figure 3, at the beginning of each trial, a fixation appeared as a cue for 3,000 ms on the black screen, followed by the pre-task. After completing the pre-task, the participant and the confederate entered the main session to divide a reward. In the *determined* condition, the participant had to make a two-alternative forced choice of whether to send (accept) an offer, which was presented by the program in the name of initialization and varied between seven Allocations to the matched person (Allocation Stage and Alternative Choice). If the participant accepted the presented offer, then each player received the payoff assigned by this offer. If he rejected sending the presented offer, he could determine any unit of money in the range of 1–9 to send to his match (Send to Others). The *predetermined* condition was similar to that in Experiment One; a program-generated offer, which was one of the seven Allocations, was presented first (Allocation Stage). Then, participants were asked to indicate whether they were satisfied with the offer (Alternative Judgment). In both conditions, the Allocation Stage lasted for 5000 ms. Afterwards, the feedback Stage lasted for 3,000 ms before the end of each trial.

All of the inequitable offers were repeated twice, and equitable offers were repeated three times. The presentation order of all trials was counterbalanced. Before the formal session, participants joined a practice session. Stimuli, recording triggers, and responses were presented adopting E-Prime 1.0 software package (Psychology Software Tools, Pittsburgh, PA, USA).

#### Skin conductance recording

While the participants were involved in the task, SCRs were continuously recorded using a BIOPAC MP150 system (Biopac Systems Inc., Goleta, CA) acquiring data at 1,000 samples per second in another computer. SCRs were recorded using two grounded Ag-AgCl electrodes (BIOPAC TSD203 transducer) that were secured medially on the distal ring and index finger of the non-dominant hand, with BIOPAC SCR paste (with a NaCl concentration of 0.05 m) as the electrolyte. Values of SCRs were baseline corrected and transformed to microsiemens (μS) values using AcqKnowledge 4.3 software. SCR amplitudes were quantified as the maximum positive change between 1 and 5 s after the start of the Allocation Stage, excluding data that did not exceed a threshold of 0.02 μs (van't Wout et al., 2006; Benedek and Kaernbach, 2010)^3^. Before each Allocation Stage, we also set a time of 6,000 ms to buffer the SCRs and made the values regress to baseline. To normalize the data, a square transformation was used, as Dunn et al. (2012) suggested.

### Results

Statistical analysis was conducted in SPSS 23.0. The Greenhouse-Geisser correction for violation of the assumption of sphericity was applied when necessary. The Bonferroni correction was used for pairwise comparisons of behavior results, while Fisher's least significant difference (LSD) test was used for SCR results^4^. Gender had no effect on behavior data [*F*_(1, 27)_ = 1.214, *p* = 0.280] or on SCR data [*F*_(1, 25)_ = 0.124, *p* = 0.728].

#### Behavior results

In the *determined* condition, the overall acceptance rates for advantageous, equitable and disadvantageous offers were 64.44% (± 38.25%), 91.11% (± 19.79%), and 58.89% (± 40.76%), respectively. A repeated measure ANOVA revealed a significant effect of Allocation [*F*_(2, 28)_ = 7.164, *p* < 0.01, η^2^ = 0.338], with equitable offers receiving more favorable responses than both advantageous (*p* < 0.01) and disadvantageous (*p* < 0.01) offers. However, the latter two had no differences between each other (*p* = 0.591). In the *predetermined* condition, the overall satisfaction rates for advantageous, equitable and disadvantageous offers were 81.25% (± 8.72%), 93.75% (± 3.36%), and 55.21% (± 11.15%), respectively. There was also a significant effect of Allocation [*F*_(2, 30)_ = 8.48, *p* < 0.001, η^2^ = 0.361], with preferences for advantageous offers being the same as those for equitable offers (*p* = 0.158), but the preferences for both of them were significantly higher than those for disadvantageous offers (*p* = 0.020 and *p* = 0.002, respectively), which was in accordance with the finding of Experiment One.

#### Skin conductance response results

SCRs elicited by each Allocation across Conditions are presented in Table 2. The repeated measure ANOVA for SCRs yielded a main effect of Allocation [*F*_(4, 109)_ = 6.482, *p* < 0.001, η^2^ = 0.194] but did not find a main effect of Condition [*F*_(1, 27)_ = 1.813, *p* = 0.189]. However, the interaction effect of Allocation and Condition was significant [*F*_(4, 109)_ = 2.744, *p* < 0.05, η^2^ = 0.092]. We adopted a simple effect analysis for Condition and found that only (5, 5) and (5, 7) produced different SCRs between Conditions [*F*_(1, 27)_ = 7.48, *p* < 0.05 and *F*_(1, 27)_ = 7.71, *p* < 0.05, respectively], with both SCRs being greater in the *predetermined* condition (vs. *determined* condition). However, SCRs elicited by the other offers had no significant differences. More importantly, we further adopted a simple effect analysis for Allocation, which showed that the effect of Allocation on SCRs was significant in both the *determined* [*F*_(6, 162)_ = 3.53, *p* < 0.01] and the *predetermined* conditions [*F*_(6, 162)_ = 5.77, *p* < 0.001]. In the following, thus, we examine this effect in the two Conditions separately.

**Table 2:** The descriptive data of SCRs elicited by each Allocation in the *determined* and *predetermined* conditions.

**Inequity type**	**Allocations**	**SCRs in the *determined* condition (μS) (*N* = 14)**	**SCRs in the *predetermined* condition (μS) (*N* = 15)**
Advantageous	(5,1)	0.2433 ± 0.9748	0.2556 ± 0.1530
	(5,2)	0.3029 ± 0.1175	0.2425 ± 0.0681
	(5,3)	0.2266 ± 0.0713	0.2361 ± 0.0987
Equitable	(5,5)	0.2017 ± 0.0539	0.2687 ± 0.0753
Disadvantageous	(5,7)	0.2197 ± 0.0793	0.3372 ± 0.1385
	(5,8)	0.2971 ± 0.1342	0.3513 ± 0.1332
	(5,9)	0.3146 ± 0.1348	0.3710 ± 0.1404

*Values of SCR were baseline corrected and transformed to microsiemens (μS) values. To normalize the data, the square transformation was used, as Dunn et al. (2012) suggested*.

**Figure 4:**
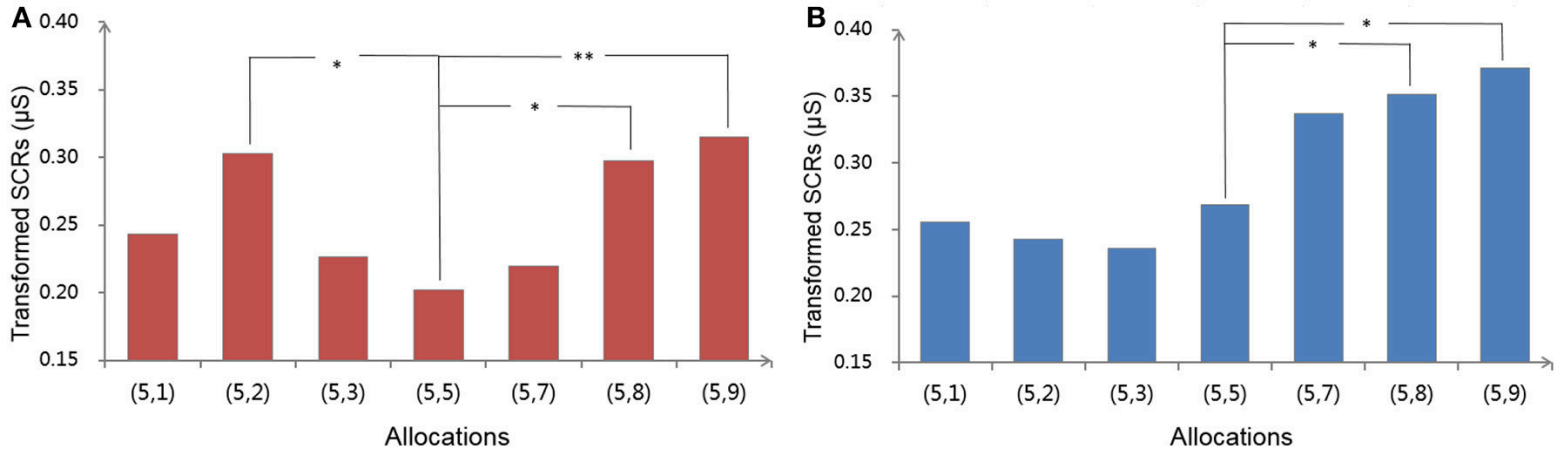
Effects of Allocation on SCRs in the *determined* (left, **A**) and *predetermined* conditions (right, **B**). Significant differences (*p* < 0.05, *p* < 0.01) between Allocations are marked with ^*^, ^**^ respectively.

In the *determined* condition, as shown in Figure 4A, pairwise comparisons showed that advantageous offer (5, 2) and disadvantageous offers (5, 8) and (5, 9) elicited a greater SCR than did the equitable offer (5, 5), and the SCR elicited by another advantageous offer (5, 1) was marginally greater than that elicited by the equitable one (*p* = 0.070). Although there is not a statistically significant difference, the actual SCRs of (5, 3) and (5, 7) were still higher than those of (5, 5). In addition, we also found that the SCRs to (5, 9) were higher than those to (5, 3) (*p* < 0.05), and SCRs to (5, 2) were higher than those to (5, 8) (*p* < 0.05), while the rest of the comparisons were not significant.

In contrast, in the *predetermined* condition, SCRs elicited by advantageous offers (5, 1), (5, 2), and (5, 3) had no differences from those elicited by (5, 5) (see Figure 4B). Although the difference was not significant, the actual SCRs of these advantageous offers were all lower than (5, 5). On the other side, SCRs elicited by disadvantageous offers (5, 9) and (5, 8) were significantly higher than those elicited by (5, 5), while those for the offer (5, 7) were marginally higher (*p* = 0.087). We also found that the SCRs for all of the disadvantageous offers were significantly higher than those for the advantageous offers, except for one comparison between (5, 8) and (5, 1).

### Discussion

Experiment Two replicated the outcome of Experiment One in the behavior analysis and further supported Hypothesis 2 by electrophysiological data. In the *determined* condition, the SCRs to both advantageous and disadvantageous inequity were higher than those to equity, whereas in the *predetermined* condition, the SCRs elicited by advantageous inequity had no significant differences from those elicited by equity. Since SCRs can be used as an electrophysiological indicator to evaluate the feeling of inequity/unfairness (van't Wout et al., 2006; Civai et al., 2010; Hewig et al., 2011; Dunn et al., 2012), we can infer that (1) if participants could determine allocations, they felt negatively toward the two types of inequity, which was consistent with previous studies, and (2) if participants passively received a program-generated allocation, however, they did not feel negatively toward receiving more than others and even felt more satisfied. Consequently, the individual's tendency toward AI may be modulated by their role in determining allocations and may disappear if they have no chance to determine the allocation.

## General discussion

We began this paper with the hypothesis that individuals' preferences for advantageous inequity might differ as a function of their role in determining allocations. Participants showed a far lower preference for equitable offers than for advantageous offers if they could *determine* allocations in the money distribution setting. However, when participants simply passed judgment on *predetermined* allocations, their preferences for advantageous offers were as high as those for equitable offers (Experiment One). We replicated this pattern of results in a further electrophysiological experiment. The SCR, an indicator of the feeling of inequity/unfairness, elicited by advantageous offers had no difference from that elicited by equitable offers in the *predetermined* condition, providing evidence that individuals did not feel negatively toward advantages in this situation (Experiment Two). Taken together, the present studies provided mutual corroboration from behavioral and electrophysiological data to document the dramatic impact of the *determined*/*predetermined* feature on AI and further noted that AI would disappear if the distribution paradigm was merely based on the *predetermined* feature.

It should be noted that, in the existing literature, it was not always true that individuals resisted receiving more than others. Some studies, which did not take the paradigm feature of *determined*/*predetermined* as their center, have found that participants appeared to prefer to receive more than others, rather than the opposite. For example, Moser et al. (2014) showed that in their ultimatum game individuals who played the role of responders (therefore, they could not decide how to divide an offer) were more likely to accept advantageous offers compared to equitable offers (acceptance rates: 97.9 vs. 91.4%, respectively, *p* < 0.05). The same pattern was also observed on the side of responders' rejection behavior. In a study conducted by Lamichhane et al. (2014), participants' rejection rates of advantageous offers (80–100% of the amount to participants) were as low as their rejection rates of equitable offers (40–60% of the amount to participants) (rejection rates: 6.5 vs. 11.3%, respectively, *p* > 0.05). Similar patterns can also be observed in Wu et al.'s (2012) and Albrecht et al.'s (2013) studies. Obviously, these studies were in conflict with the previous studies on AI, which claimed that people would resist receiving more than others (Fehr and Schmidt, 1999, 2006). We suggest that a clue for understanding this conflicting result can be found by investigating the paradigm feature of *determined*/*predetermined*, which is still unclear. Unlike those in previous studies, the allocations in these studies were not proposed by participants, as was the case of the *predetermined* condition in the present study. Therefore, it may have been due to the fact that their research paradigms were mainly based on the *predetermined* feature, then they failed to reveal the tendency of AI and were in conflict with previous studies. Although these studies did not take the effect of the *determined*/*predetermined* feature as their focus, they provide additional evidence to support our hypothesis that AI would diminish or even disappear if advantageous inequity is *predetermined*.

One of the potential causes for individuals' different behaviors between the *determined* and *predetermined* conditions may be the sense of agency, referring to the subjective experience of controlling one's own actions, and through these actions, controlling external events (Gallagher, 2000). Interestingly, the notion of agency in the cognitive literature refers mostly to a person's control over the outcomes of his actions (Caspar et al., 2016), which is innately related to the paradigm feature of *determined*/*predetermined*. According to Choshen-Hillel and Yaniv (2011, 2012), there is a causal relationship between the sense of agency and one's concern with others' well-being. More specifically, in settings in which people have a high agency, their concern with others' welfare is prominent, whereas in settings in which people have a low agency, their concern with self-interest (in their studies, this means avoiding receiving less than others) figures prominently. In addition, this effect even exists in children from 3 to 4 years old, with children given a sense of agency becoming happier to share more with a new individual (Chernyak and Kushnir, 2013). It is implied that individuals who have a higher agency could derive some internal rewards from being kind to others, and the gained positive utilities, in turn, could partly offset the cost of the benevolence (Choshen-Hillel and Yaniv, 2011, 2012). In our terms, the *determined* condition mostly refers to a high-agency condition, and the *predetermined* condition mostly refers to a low-agency condition. Due to the fact that the sense of agency could make people's focus change from self-interest to the other's welfare, the result that those who were in the *determined* condition were more likely to keep offers equitable than those who were in the *predetermined* condition is to be expected. We suggest that the sense of agency may serve as an approach motivation to push people in the *determined* condition to behave as the theory of inequity aversion expects. Conversely, it also provides an explanation for why AI would diminish or even disappear when people are in the *predetermined* condition. On the other hand, the responsibility for negative consequences may also play a role. In our experiments, the linkage between one's actions and outcomes was stronger in the *determined* condition than in the *predetermined* condition. The allocations of the *determined* condition were totally decided by participants, and because of this, participants needed to take responsibility for the final distribution outcomes. Nevertheless, the outcomes of the *predetermined* condition were not due to participants' actions, and thus, they were free to be responsible for the final outcomes. To date, a body of studies have demonstrated that, as the linkage between actions and outcomes becomes stronger, decision-makers would not only show greater prosocial preferences toward others (Hamman et al., 2010; Bartling and Fischbacher, 2011) but also be more in compliance with social norms (Andreoni and Gee, 2012; Kamei et al., 2014). That is, because being responsible means being blameworthy for potentially negative outcomes and the prospect of blame for immoral behaviors (e.g., selfish or greedy) would make people avoid doing the things inconsistent with social expectations (Bartling and Fischbacher, 2011). Since seeking advantageous inequity is commonly viewed as a behavior that is inconsistent with social expectations (Spitzer et al., 2007; Fershtman et al., 2012), participants in the *determined* condition would avoid showing this behavior and conversely choice to be equitable with others. Because of this, responsibility may work as an avoidance motivation to pull people in the *determined* condition to acquire advantageous inequity. In contrast, people in the *predetermined* condition may be free from blame for receiving more than others because they need not to take negative actions in the allocating. As a result, they would feel less negatively toward advantageous inequity and thus be willing to accept it.

Taken together, maybe both the approach motivation of concerning with others (sense of agency) and the avoidance motivation of avoiding blame (responsibility) work together to inhibit people's preferences for advantageous inequity in the *determined* condition. However, the *predetermined* condition is the routine case without the sense of agency and the responsibility. Individuals in this condition may be free to receive advantageous inequity. Further testing is needed to tease apart these possible interpretations of the participants' behavior.

Theoretically, there is a more in-depth discussion referring to the question of whether AI is an authentic behavioral tendency in human beings. Notice that, in the present study, the tendency of AI occurs only in the situation where participants can determine allocations, while it disappears if they cannot. If AI, according to the definition of inequity aversion, is focused on avoiding receiving more than others, why would it be observed in one condition (*determined*) and not in the other (*predetermined*)? Maybe the reactions to advantageous inequity have different psychological mechanisms between the *determined* and *predetermined* conditions. Alternatively, maybe the tendency of AI does not have a solid foundation, but other motivations are preventing individuals from showing satisfaction for advantageous inequity in the *determined* condition. This question needs to be examined in future works.

To our knowledge, the present study might be the first to investigate correlates of the impact of individuals' role in determining allocations on their preferences for advantageous inequity and to prove that if individuals cannot determine allocations, their tendency of AI would disappear. The present study has at least three contributions to the current research. First, our questions directly concern the structural integrity of the theory of inequity aversion (especially on advantageous inequity) and help extend the current research from the *determined* domain to the *predetermined* domain. If we make the paradigm feature (*determined* vs. *predetermined*) and types of inequity aversion (AI vs. DI) intersect with each other, we can get four connections: *determined*—AI, *predetermined*—AI, *determined*—DI, and *predetermined*—DI. The first and the fourth connections are used as the general method to investigate AI and DI by the current researches (Sanfey et al., 2003; Fehr and Schmidt, 2006; Fehr et al., 2008), and the third connection is commonly regarded as a form of the altruism preference (Batson and Powell, 2003), leaving the second connection still unclear. As shown, the present study sheds light on this gap, suggesting that the tendency of AI is weak in this connection. Second, the present study reveals that AI has a boundary condition: only those who are in the *determined* condition would show AI, whereas those who are in the *predetermined* condition would not. This further implies that the current literature related to AI might be biased because almost all of the studies are based only on the *determined* feature, ignoring the situation of the *predetermined* feature. Thus, caution should be used in generalizing to other situations the conclusion that individuals resist advantageous inequity. Further studies, however, are required to explore the mechanism behind this boundary effect. Third, the present finding is important methodologically because it may help reconcile why most of the past studies found a robust tendency for humans to resist advantageous inequity (Fehr and Schmidt, 1999, 2006), whereas another group of studies mentioned above instead demonstrated that individuals are happy to receive advantageous inequity (Wu et al., 2012; Albrecht et al., 2013; Lamichhane et al., 2014; Moser et al., 2014). We suggest that differences concerning the *determined*/*predetermined* feature cause these ostensible conflicts. Thus, the existent conflicting results can be unified into a common theoretical framework by the present study.

One limitation of the present study is that we carried out comparisons between choice (as the dependent variable of the *determined* condition) and satisfaction (as the dependent variable of the *predetermined* condition). As mentioned above, the revealed preference theory implied that one would choose the thing that satisfies him most (Samuelson, 1938). In this case, to a certain extent, the choice that people makes is whatever they are content with, thus establishing a connection between choice and satisfaction. Actually, in two studies conducted by Choshen-Hillel and Yaniv (2011, 2012), conditions were very similar to that of the present study, and they also carried out direct comparison between choice and satisfaction. Similarly, in the field of equity aversion, researchers often make direct comparisons between and conduct subsequent discussions on AI and DI (noting that AI is based on choice and DI is based on satisfaction) (Güth and Kocher, 2014; Tricomi and Sullivan-Toole, 2015; Xu et al., 2016). Although there are reasonable arguments for comparing choice and satisfaction, it does indeed have its limits. Therefore, further works should develop a better research paradigm to overcome the problem of direct comparisons.

## Conclusion

The current study demonstrated that individual's tendency of AI might differ as a function of their role in determining allocations. Both behavioral and electrophysiological data showed that, in the situation in which participants could determine allocations, they seemed to dislike advantageous inequity, which is consistent with the prediction of the theory of inequity aversion. However, in the situation in which participants could not determine allocations, they appeared to prefer advantageous inequity. This finding suggests the possibility that the tendency of AI may have a different mechanism between the two situations, or more strictly, it does not have a solid foundation, and the preference for advantageous inequity that would exist in the *determined* condition may have been prevented by other factors.

## Ethics statement

This study was carried out in accordance with the requirements of the Ethics Committee at Central China Normal University. All subjects gave a written informed consent according to the Declaration of Helsinki. The protocol was approved by the Ethics Committee at Central China Normal University.

## Author contributions

OL had made contributions to the conception, design, analysis, acquisition and interpretation of the data. OL also wrote the first manuscript. LW and FX revised the manuscript and gave excellent advice. OL, LW, and FX finally approved the version to be submitted.

### Conflict of interest statement

The authors declare that the research was conducted in the absence of any commercial or financial relationships that could be construed as a potential conflict of interest. The handling Editor declared a shared affiliation, though no other collaboration, with the authors OL and LW.

## Footnotes

^1^A routine way is to use the ultimatum game (Güth et al., 1982) or its modified version, the dictator game (Kahneman et al., 1986), as the design, in which participants act as the role of proposer who can divide the offer by himself (Fehr and Schmidt, 2006).

^2^The research objects of these studies are various. Albrecht et al. (2013) focused on the effect of status on satisfaction with relative rewards; Moser et al. (2014) focused on how social information and personal interests change fairness in decision-making; and Lamichhane et al. (2014) focused on the comparison of the neural basis between advantageous and disadvantageous inequity.

^3^Participants displayed SCRs to 12.28 out of 15 trials on average (mean nonresponses = 2.72 ± 2.90). To examine whether the proportion of nonresponses varied as a function of Allocation, two Cochran's Q tests were conducted, showing that nonresponses were evenly distributed (for the *determined* condition, χ^2^ = 1.714, *p* = 0.424; for the *predetermined* condition, χ^2^ = 5.750, *p* = 0.452). A further M-W U test showed that nonresponse rates were relatively comparable across Conditions (Z = 1.680, *p* = 0.092).

^4^We did not use Bonferroni correction to correct the *p*-value of multiple comparisons for SCRs data because it made almost all of the comparisons non-salient. The within-factor of Allocation had 7 levels, which required carrying out 21 comparisons to complete the *post-hoc* test. This would make the corrected significance level too low (i.e., 0.05/21 = 0.00238) to be detected and then increase the likelihood of making a β-error. Actually, the data difference between experimental treatments in the SCR tasks was not as obvious as that in the behavior tasks; thus, the actual effect might be covered under such a low significance level if we applied this correction.

## Appendix A

### The analyses incorporated filler tasks of (6, 4) and (4, 6)

Statistics analysis was conducted in SPSS 23.0. The Greenhouse-Geisser correction for violation of the assumption of sphericity was applied when necessary. The Bonferroni correction was used for pairwise comparisons.

The preference for (6, 4) and (4, 6) were 15.25 and 8.47%, respectively. After incorporated (6, 4) and (4, 6), the main effects of Condition [*F*_(1, 116)_ = 757.5, *p* < 0.001, η^2^ = 0.867] and Allocation [*F*_(3, 381)_ = 110.2, *p* < 0.001, η^2^ = 0.487] were still significant, the same as the interaction effect of the two factors [*F*_(3, 381)_ = 35.7, *p* < 0.001, η^2^ = 0.235]. The results of simple effect analyses for Allocation showed that this factor still had a significant effect in both the *determined* [*F*_(6, 696)_ = 68.62, *p* < 0.001] and *predetermined* [*F*_(6, 696)_ = 77.29, *p* < 0.001] conditions. Considering to (6, 4) and (4, 6), although they were the lower magnitude (dis)advantageous inequity, participants were still less likely to choose them compared to (5, 5) (*p* < 0.001 for both) in the *determined* condition. In contrast, in the *predetermined* condition, the preference for (6, 4) was not different from (5, 5) (*p* > 0.05), while both of them were significantly higher than (4, 6) (*p* < 0.001 for both).

From these results, we can find that statistical trend for (6, 4) and (4, 6) were the same as that for other advantageous and disadvantageous offers, respectively, in the Experiment One. Even if we incorporated (6, 4) and (4, 6) into analysis, the results remained unchanged.
